# Electrophysiological and Structural Remodeling in Heart Failure Modulate Arrhythmogenesis. 2D Simulation Study

**DOI:** 10.1371/journal.pone.0103273

**Published:** 2014-07-23

**Authors:** Juan F. Gomez, Karen Cardona, Laura Martinez, Javier Saiz, Beatriz Trenor

**Affiliations:** Instituto de Investigación en Ingeniería Biomédica, UniversitatPolitècnica de València, Valencia, Spain; Gent University, Belgium

## Abstract

**Background:**

Heart failure is operationally defined as the inability of the heart to maintain blood flow to meet the needs of the body and it is the final common pathway of various cardiac pathologies. Electrophysiological remodeling, intercellular uncoupling and a pro-fibrotic response have been identified as major arrhythmogenic factors in heart failure.

**Objective:**

In this study we investigate vulnerability to reentry under heart failure conditions by incorporating established electrophysiological and anatomical remodeling using computer simulations.

**Methods:**

The electrical activity of human transmural ventricular tissue (5 cm×5 cm) was simulated using the human ventricular action potential model Grandi et al. under control and heart failure conditions. The MacCannell et al. model was used to model fibroblast electrical activity, and their electrotonic interactions with myocytes. Selected degrees of diffuse fibrosis and variations in intercellular coupling were considered and the vulnerable window (VW) for reentry was evaluated following cross-field stimulation.

**Results:**

No reentry was observed in normal conditions or in the presence of HF ionic remodeling. However, defined amount of fibrosis and/or cellular uncoupling were sufficient to elicit reentrant activity. Under conditions where reentry was generated, HF electrophysiological remodeling did not alter the width of the VW. However, intermediate fibrosis and cellular uncoupling significantly widened the VW. In addition, biphasic behavior was observed, as very high fibrotic content or very low tissue conductivity hampered the development of reentry. Detailed phase analysis of reentry dynamics revealed an increase of phase singularities with progressive fibrotic components.

**Conclusion:**

Structural remodeling is a key factor in the genesis of vulnerability to reentry. A range of intermediate levels of fibrosis and intercellular uncoupling can combine to favor reentrant activity.

## Introduction

Ventricular arrhythmias in patients with congestive heart failure (HF), contribute to the high incidence of sudden cardiac death associated with HF [1], [2]. The mechanisms of the arrhythmias occurring in the setting of HF are not fully understood. Afterdepolarization-induced trigger activity has a high tendency to develop in the failing myocardium. However, conditions favoring reentrant arrhythmias have also been described in failing hearts [2]. Reentrant activity is generated by wave interaction with anatomical or functional obstacles combined with specific excitability conditions [3]. In diseased hearts, preexisting electro anatomic tissue heterogeneity is amplified considerably, increasing vulnerability to reentrant arrhythmias [3], [4].

In the case of HF, electrical and structural changes significantly increase the occurrence of reentry. The failing heart phenotype is characterized by distinct alterations in selected ion channels, changes in intracellular calcium cycling, alterations in cell-cell coupling proteins, enhanced interstitial fibrosis, and cellular hypertrophy [5]–[7]. Experimental studies in animal models provide evidence for enhanced repolarization gradients in the setting of the failing heart [8], [9] and these can promote reentrant arrhythmias. However, recent studies performed using explanted failing human hearts [6], [10], [11] provide little evidence for these enhanced repolarization gradients. These controversial results were theoretically approached in our accompanying paper [12], showing that heterogeneous ionic remodeling modulates repolarization gradients. These repolarization gradients can also be enhanced by structural remodeling, for example fibrosis and/or cellular uncoupling [13], which simultaneously alter conduction properties [11], [14], [15], increasing the likelihood of reentry. The lack of detailed functional and structural information limits the utility of experimental studies for identifying the precise role of HF remodeling on propagation disorders of the cardiac electrical activity. Computational approach can provide a powerful tool for the analysis of the contributions of different components of a disease. Simulations of reentrant rhythms in the human heart with emphasis on electrophysiological remodeling in HF have been published by Moreno et al. [16] focussing on the effect of drugs. Zlochiver et al. [17] evaluated the current density threshold for cardiac resynchronization treatment, and Turner et al. [18] analyzed electrogram fractionation. Recent studies have simulated the effects of fibrosis on reentry in ventricle [4], [17], [19]–[23] and atrium [24], [25]. However, no simulation studies combining electrophysiological HF remodeling, fibrosis and intercellular uncoupling to analyze the vulnerability to reentry in the failing human heart have been performed (see Figure S1, Figure S2, Figure S3, and Figure S4 in File S1). Furthermore, the analysis of the reentry dynamics under these pathological conditions has not been addressed. We have evaluated whether phase analysis could reveal how heterogeneities caused by electrophysiological and structural remodeling may lead to reentrant waves. Indeed, phase analysis provides a useful tool to follow the electrical propagation activity of the spiral wave and to analyze the arrhythmogenic substrate under such pathophysiological settings. Several studies have used phase maps to follow the trajectory of reentrant activity experimentally [26], [27] and theoretically [28], [29] but none of these have focused on considered conditions of heart failure remodeling in conjunction with fibrosis.

In the present study the electrical activity of a transmural two-dimensional human ventricular tissue was simulated using a human action potential (AP) model [30], [31] modified to replicate a HF phenotype. The influence of HF-induced electrophysiological and structural remodeling on vulnerability for reentrant arrhythmia occurrence was studied, and the dynamics of reentrant circuits under such pathological conditions were analyzed.

## Methods

### Tansmural failing tissue

The electrical activity of a two dimensional (2D) cardiac tissue of 5 cm×5 cm was simulated. Anisotropy was modeled by considering that transverse conductivity was lower than longitudinal conductivity (anisotropy conductance ratio 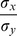 = 4), and the longitudinal direction of cellular fibers was horizontal. Half of the tissue corresponded to endocardial cells and the other half to epicardial cells. This domain was discretized with 250000 square elements (Δx = 0.01 cm) which resulted in a total of 251001 nodes within the tissue. Temporal resolution (Δt) was fixed to 0.002 ms to ensure numerical convergence (Δt<Δx^2^/2D). Human myocyte electrical activity was simulated using a modified version of Grandi et al. model (GPB) [30], in which a late sodium current (I_NaL_) was added [31]. In order to assess the influence of HF-induced electrophysiological remodeling, our HF model [31] was implemented, as described in the accompanying paper [12]. Thereafter, structural remodeling was also considered by adding ‘fibrosis’ and/or intercellular uncoupling within this tissue. The electrical activity of fibroblasts was simulated using the ‘active’ formulation of MacCannell et al. [32].

The diffusion coefficient (D) in normal conditions (NC) for myocytes was set to D_M_ = 0.0013 cm^2^/ms resulting in a conduction velocity (CV) of 50 cm/s which agreed with experimental measurements of transmural conduction [33]. To simulate cell-to-cell uncoupling in heart failure, D was reduced in accordance with experimental data [34]. A 50% reduction was applied between failing myocytes (D_LOW_) and a three-fold reduction was used between fibroblasts [35], [36] and for the myocyte-fibroblast interaction (D_Fib_). A wide range of fibroblast-myocyte gap-junctional conductance has been reported in experimental [35], [37], [38] and simulation studies [4], [32], [36], [39], [40]. Selected values within these ranges were used.

Fibroblast distribution was organized randomly, by assigning a probabilistic function. Random arrays were generated with different probabilities of assigning the fibroblast ionic model to the nodes. Four configurations of the same random condition were made. These gave rise to different levels of fibrosis quantified as the percentage of nodes executing the fibroblast model (P_f_). Tissue configurations were denoted as “minimal fibrosis” (P_f_ = 4%), “mild fibrosis 1” (P_f_ = 14.5%), “mild fibrosis 2” (P_f_ = 28%), and “high fibrosis” (P_f_ = 40%). These values of P_f_ are similar to the ones considered in [20] and would correspond to fibrotic areas within experimental ranges [41].

**Figure 1:**
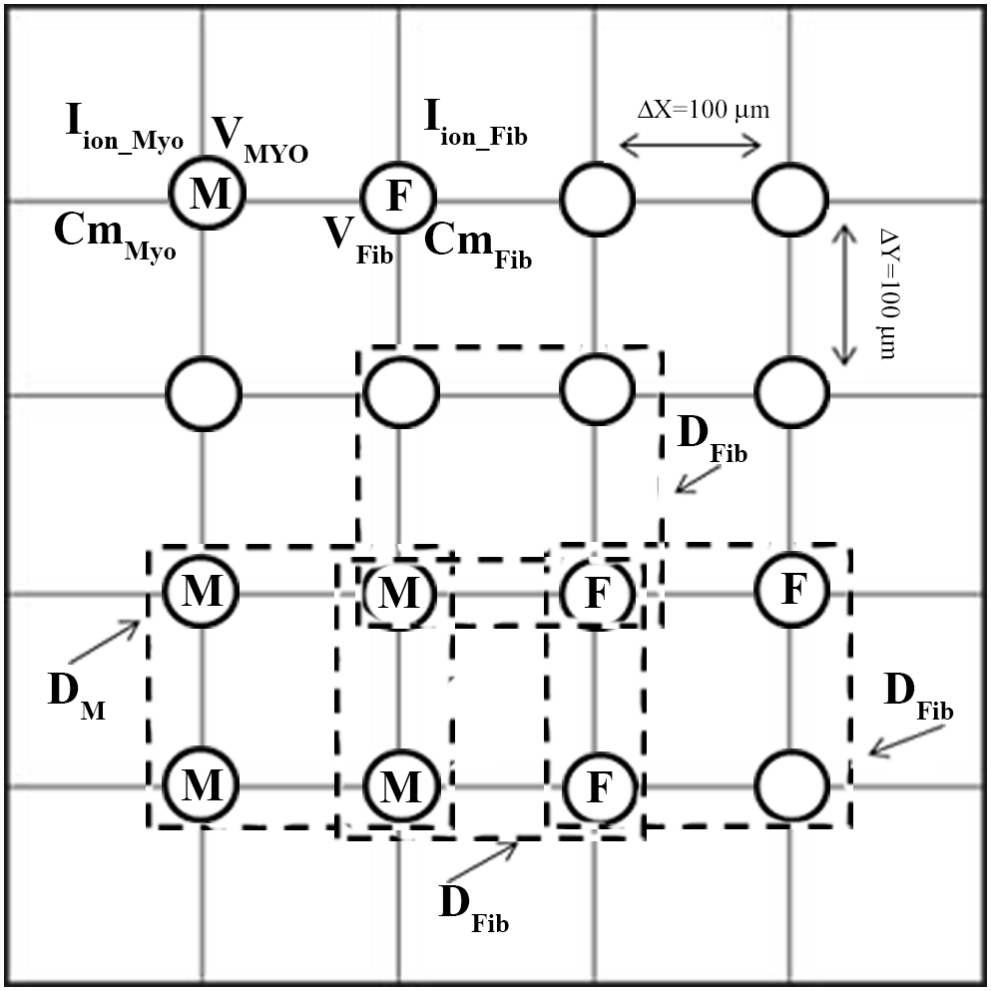
Illustration of the configuration of our virtual human ventricular mesh of interconnected myocytes and fibroblasts. V is the membrane voltage computed for each node, i.e. V_MYO_ for myocytes and V_Fib_ for fibroblasts. 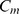 is the cell capacitance, i.e. C_mMyo_ for myocytes and C_mFib_ for fibroblasts. D is the diffusion coefficient, i.e. D_M_ between myocytes in normal conditions (D_M_ = 0.0013 cm^2^/ms), and D_Fib_ between a myocyte and a fibroblast, assigned to each element in the discretized domain. 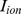 is the total transmembrane ionic current, i.e. I_MYO_ for myocytes and I_Fib_ for fibroblasts. ‘M’ and ‘F’ correspond to the ‘myocyte’ and ‘fibroblast’ model respectively that are computed in each node.

As stated above, the mesh was discretized in square elements of 100 µm×100 µm. One element was composed of four nodes (separated in 100 µm), as shown in Figure 1. Each node executed either the fibroblast ionic model or the myocyte AP model. The value of the diffusion coefficient of a myocyte (D_M_) was assigned to an element composed of 4 nodes executing the myocyte AP model. If one or more nodes of an element executed the fibroblast ionic model, then the diffusion coefficient of a fibroblast (D_Fib_), was assigned to this element.

### Stimulation Protocol

After achieving steady-state conditions, five stimuli adjusted to 2 times the excitation threshold in amplitude and 2 ms in duration were applied at the endocardial end of the tissue with a basic cycle length (BCL) of 1000 ms. Electrophysiological properties were measured for the last AP.

To generate spiral wave in 2D simulations, we applied the cross-field stimulation protocol [42]. Once the tissue was stabilized, five stimuli were applied at the endocardial end of the tissue to get an entire wavefront throughout the tissue. After allowing the excitation wave-tail corresponding to the last stimulus to reach the middle part of the tissue, a second S2 stimulation was applied at the endocardial left bottom corner of the tissue (2.5 cm high×1.25 cm wide). This triggered a meandering activation wave (due to wavefront/wave-tail interaction) leading to the formation of a spiral wave. The vulnerable window (VW), an indicator for vulnerability to reentry, was defined as the value of the coupling intervals (CIs) for S2 that led to reentry (at least two spiral wave rotations).

### Spiral Wave Dynamics

To analyze the dynamics of the spiral waves, the dominant spiral wave period was calculated and phase analyses were performed.

Using the information of the AP upstroke in all points of the sheet, histograms of cycle lengths values measured to the nearest 1 ms were developed during the simulation and were used to calculate the dominant spiral wave period as in [43].

Phase analysis of the membrane potential signal (V_m_) represents a useful method to identify and quantify spatiotemporal organization of reentry dynamics. The phase tracks the progression of a defined region of the myocardium through the action potential and has been demonstrated to be an effective parameter in analyzing spatiotemporal changes. Points around which the phase progresses through a complete cycle from – π to π are of special interest. At these points, the phase becomes indeterminate and the activation wave fronts hinge on these points and rotate around them in an organized fashion. These points in the phase map are called phase singularity points (PS). PSs share location with anatomic heterogeneities, and their spatial meandering is modulated by these heterogeneities. PS also correlate with the location of wave breaks [44] in myopathic human hearts. At a phase singularity point the following condition is met:
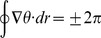
(1)


where 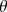 is the instantaneous phase. The integral is computed along the closed curve surrounding the anatomical or functional defect.

To obtain phase maps an algorithm in MATLAB calculated the Hilbert Transform (HT) of the membrane potential (V_m_) signal in every node of the discretized domain to avoid the results dependence with the chosen time delay [26], and the ‘instantaneous phase’ was calculated in each node as follows:
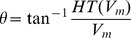
(2)


**Figure 2:**
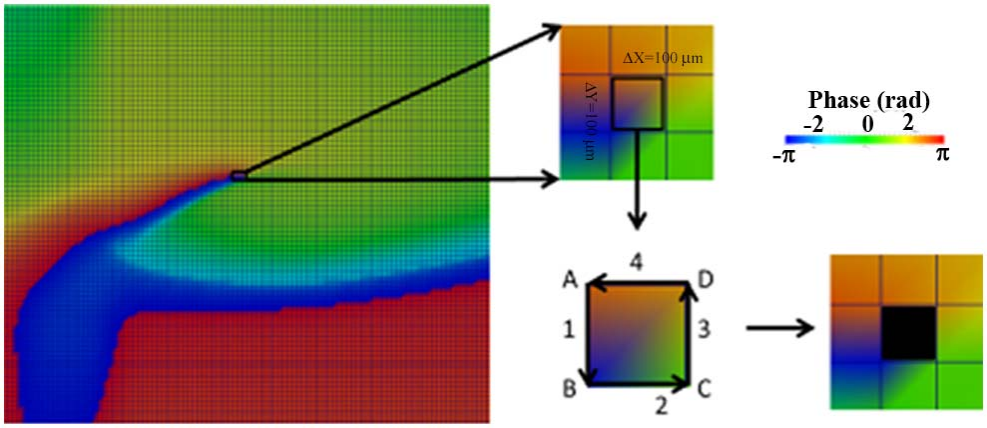
Illustration of phase map analysis. Instantaneous phase of the membrane potential (V_m_) signal at the different nodes of the tissue is computed. A phase singularity in an element is operationally defined as all phases being detected within the element (see Methods section for details).

Phase maps were codified between [–π, π] as in [45]. In our discretized mesh domain PS was defined as a singular point around which the condition of equation (3) was met.
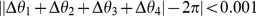
(3)


where 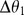 is the phase difference between nodes B and A, 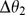 is the phase difference between nodes C and B, 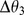 is the phase difference between nodes D and C, 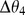 is the phase difference between nodes A and D [45], as depicted in Figure 2.

### Computational methods

ELVIRA software was used in our simulations, which is based on a pseudo-adaptive finite element method in space and time to solve reaction–diffusion equations with highly nonlinear reactive terms [46]. The scheme accounts for the anisotropy of the media and incorporates an adaptive time step algorithm to integrate the stiff reactive term associated with the ionic currents. The solution of the monodomain equation (4) is computed using the technique of operator splitting.
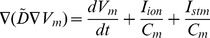
(4)

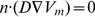
(5)


Where V_m_ is membrane potential in (V), 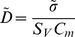 in (m^2^/s) is the diffusion conductivity tensor, 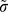 is the conductivity tensor in (S.m), 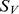 is the surface to volume ratio of the cell in (m^−1^), 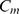 in (F) is the membrane capacity, 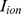 is the ionic total current in (A), and 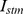 in (A) is the stimulus current. Equation (4) has boundary conditions (5). Further information will be found in [46]. Tissue mesh representation is shown in Figure 1.

## Results

### Effects of heart failure remodeling on reentry generation

To simulate reentrant activity, cross-field stimulation was applied to the transmural tissue composed of endocardial and epicardial cells, as described in the Methods section. Reentrant activity or unidirectional block was not observed, either in normal conditions or under HF ionic remodeling conditions. In both situations repolarization time was too large to enable propagation and the wave front was blocked after premature stimulation. If the premature stimulus S2 was applied with a longer coupling interval (S1–S2), complete propagation of S2 was achieved. It is to be noted that the size of the tissue, 5 cm×5 cm in the present simulations, is an important factor for the generation of spiral waves [47], and bigger tissues might lead to the generation of reentry for the above conditions. However, because of the high computational cost of a larger tissue, simulations were performed for the described size.

**Figure 3:**
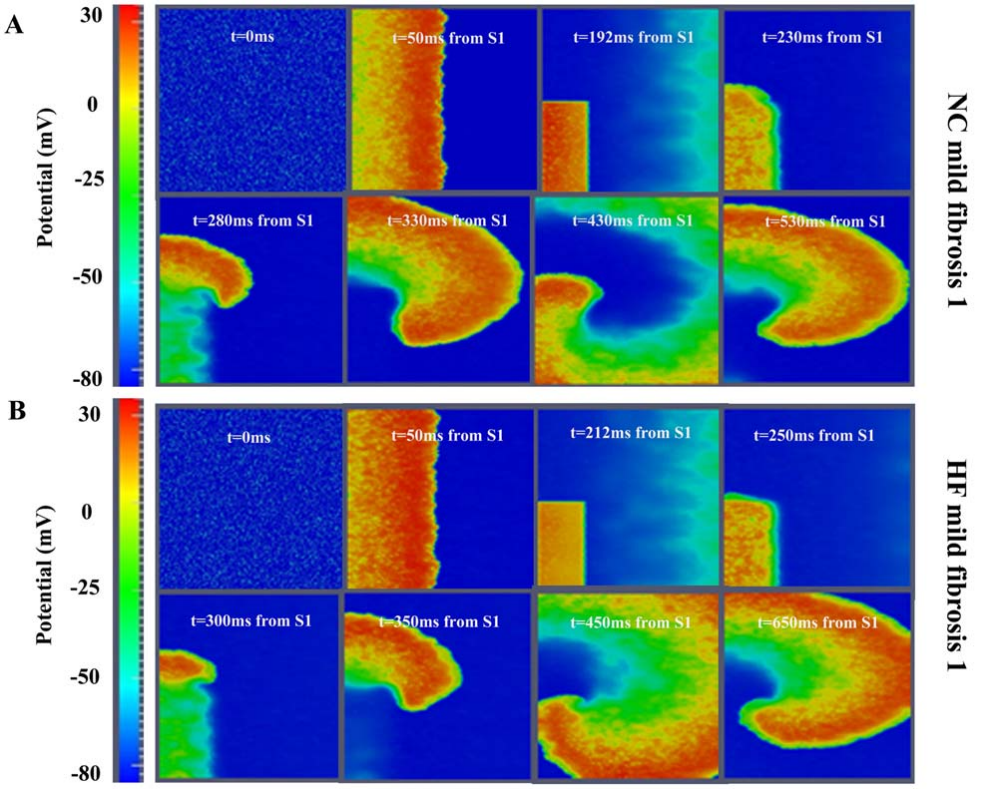
Reentrant activity intransmural fibrotic human ventricular tissue. A. Voltage snapshots in the transmural tissue under normal conditions (NC) and following introduction of 14.5% random fibrosis (mildfibrosis1). B. Voltage snapshots in the transmural tissue assuming heart failure (HF) induced electrophysiological remodeling together with 14.5% random fibrosis (mildfibrosis1). In both panels a cross-field protocol S1–S2 was applied to initiate conduction (see Methods section for details). For full spatiotemporal evolution of panel A see **Video S1**.

Fibrosis (14.5%) was then added to NC configuration and reentrant activity was observed (see Figure 3, panel A). The spatiotemporal evolution of the reentrant wave is shown in Video S1. A set of simulations varying the coupling interval was performed to determine the vulnerable window for reentry (VW). This was approximately 20 ms. When the HF model was studied with the same amount of fibrosis, reentrant activity was also observed (see Figure 3, panel B). The VW was as wide as that in NC, but the limits of the VW were delayed (corresponding to bigger coupling intervals).

These results suggest that HF electrical remodeling does not alter the width of the VW, and for this reason the likelihood of reentrant activity is unchanged. However, fibrosis seems to be an important determining factor for reentry generation.

### Effects of fibrosis on reentry generation

**Figure 4:**
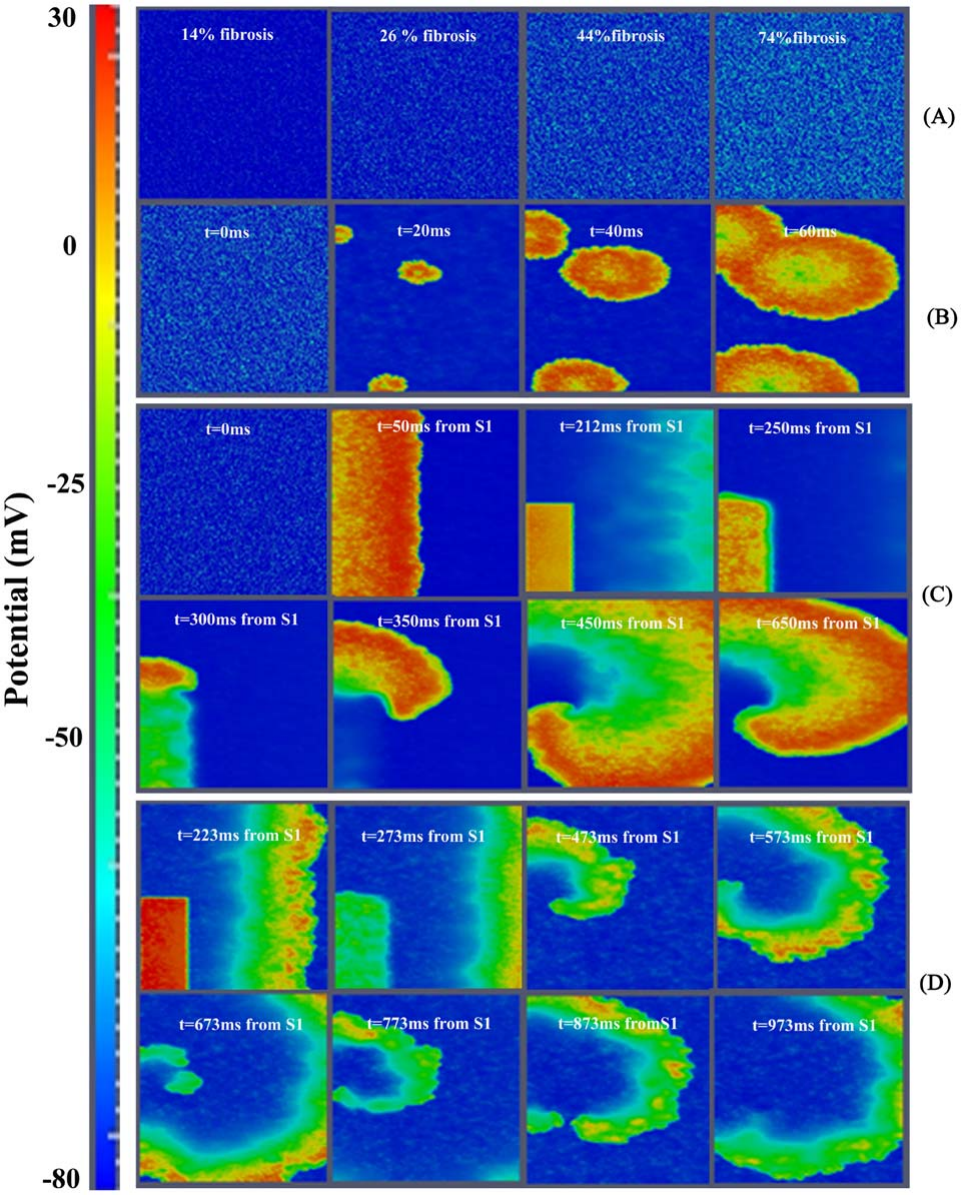
Illustration of the effect of fibrosis in human ventricular tissue. A. Voltage snapshots corresponding to initial conditions, different fibrotic tissue configurations. B. Spontaneous electrical activity in ‘mildfibrosis 2’. C. Voltage snapshots of transmural tissue assuming heart failure conditions together with 28% of random fibrosis (‘mildfibrosis 1’). In the first two frames, propagation of the action potential due to the S1 stimulus is observed and note also that propagation of the action potential elucidated by the S2 stimulus is observed after the third frame. D. Voltage snapshots of transmural tissue assuming heart failure conditions together with 40% of random fibrosis (‘high fibrosis’). S1–S2 cross-field protocol was also used to stimulate the tissue, but in this case only the propagation of stimulus S2 is shown. For full spatiotemporal evolution of panel B see **Video S2**. For full spatiotemporal evolution of panel D see **Video S3**.

In order to understand the role of fibrosis in reentry generation several degrees of fibrosis were considered. Figure 4 panel A shows the initial membrane potential for different densities of fibroblasts in the failing tissue. Due to the depolarized resting potential of fibroblasts, spontaneous activity appeared in different zones of the ventricular tissue even when the percentage of fibrosis was relatively small (mild fibrosis 2), as shown in Figure 4 panel B (ectopic activity shown in Video S2). After cross-field stimulation, as described above, the presence of intermediate fibrosis (‘mild fibrosis 1’, 14.5% of fibrotic content) led to reentrant activity in both normal and HF conditions. A more detailed analysis of how fibrosis density can affect the probability of reentry was carried out in HF conditions, (when fibrosis is known to develop). Indeed, the quantity of fibrosis significantly alters the electrical properties of the tissue, as we have shown in the accompanying paper [12]. When ‘low fibrosis’ (4%) configuration was considered, we could not induce reentrant activity, regardless of the CI. Thus, there was no VW. As described in the previous section, with an intermediate level of fibrosis (14.5%) a VW of around 20 ms was obtained (see Table 1). Indeed, when enough fibrosis is present, the APD and effective refractory period of the myocardial cells are shortened [12], so that when the premature stimulation was applied, part of the ventricular tissue had recovered enough to be excited and a spiral reentry was generated (see Figure 3 panel B). Similar results were obtained using ‘mild-fibrosis 2’ (28%) configuration, and an example of reentry is shown in Figure 4 panel C. Under these conditions, the VW was even longer, around 33 ms. When the ‘high fibrosis’ (40%) configuration was evaluated (see Figure 4 panel D), a spiral wave front could be observed. However, in this case, the depolarizing wave front reached very low potentials, leading to electrotonic voltage changes (the spatiotemporal evolution of the wave is shown in Video S3), and could not be considered as reentrant activity, as will be demonstrated later in the phase maps analysis. Thus, intermediate fibrosis increases the VW and very high fibrosis avoids reentry generation, as summarized in Table 1. Several random realizations were simulated for each degree of fibrosis (see Table S1 in File S1) and similar results were obtained for the widths of the vulnerable windows corresponding to the same amount of fibrosis.

**Table 1:** Vulnerable window analysis.

Simulation	Vulnerable window for reentry (ms)	Limits of the VW (ms)	RotationPeriod (ms)
HF 4% fibrosis	0	-	0
HF 14.5% fibrosis	20	[204;224]	240
HF 28% fibrosis	33	[170;203]	250
HF 40% fibrosis	0	-	0

**Figure 5:**
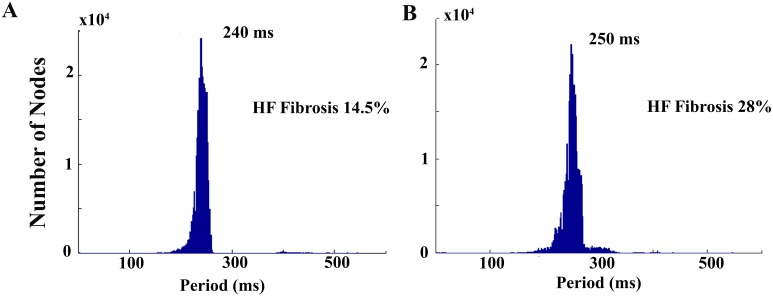
Spiral waves periods. Using the information of the AP upstroke in all points of the sheet, histograms of cycle lengths values measured to the nearest 1[43]. The reentrant waves generated after an S2 stimulus applied at an instant of time situated in the center of the vulnerable window (S2 applied at t = 210 ms for mild fibrosis 1 and t = 170 ms for mild fibrosis 2) were considered to build the histograms. The dominant spiral wave periods were 240 ms and 250 ms for mild fibrosis 1 and 2, respectively.

Vulnerable window for reentry under conditions of heart failure (HF) induced ionic remodeling with different amounts of diffuse fibrosis. The first column shows the width of the vulnerable window, the second column shows the limits of the vulnerable windows, i.e. the first and last instants of time at which S2 is applied to generate reentry, the third column shows the rotation period of the spiral waves taken from Figure 5.

In the cases where spiral waves were obtained the dominant period was measured, as shown in Figure 5 and indicated in Table 1. For a reentrant wave generated after an S2 stimulus applied at an instant of time within the vulnerable window (S2 applied at t = 210 ms for mild fibrosis 1 and t = 170 ms for mild fibrosis 2) the dominant spiral wave periods were 240 ms and 250 ms for mild fibrosis 1 and 2, respectively. Thus, the rotation frequency of the spiral wave slightly decreased with increased fibrosis.

### Intercellular uncoupling effects on reentry generation

**Figure 6:**
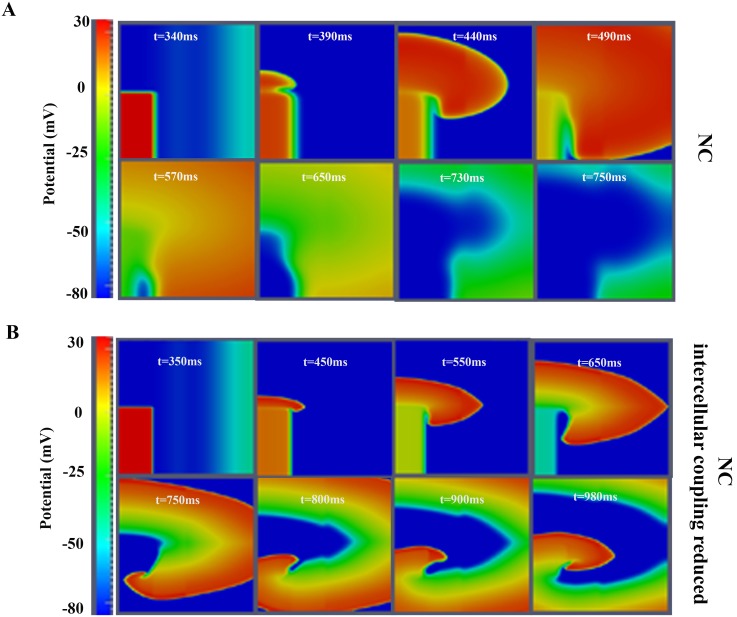
Effects of cell-to-cell uncoupling on reentry generation. A. Voltage snapshots in the transmural human ventricular tissue under normal conditions (NC). B. Voltage snapshots in the transmural tissue NC with a marked decrease in intercellular coupling (conductivity was reduced ten-fold). S1–S2 cross-field protocol was used to stimulate the tissue. Only the response to S2 stimulus is shown. For full spatiotemporal evolution of panel B see **Video S4**.

The structural remodeling of HF includes not only fibrosis but also intercellular uncoupling. To understand the role of this uncoupling in the generation of reentry, simulations were performed using NC tissue with reduced electrical conductivity (DLOW). Under normal coupling conditions, reentry was not obtained (see Figure 6 panel A) for the selected size of the tissue. Thus, the conductivity was gradually reduced until reentrant activity developed (see Figure 6 panel B), which would be formally equivalent to increase the size of the tissue. This required a ten-fold reduction of the diffusion coefficient (the spatiotemporal evolution of the reentrant wave is shown in Video S4). Normal CV was 50 cm/s [33], and was reduced down to 18 cm/s, for the low conductivity conditions. This could also be estimated by the proportionality between CV and √D.

**Figure 7:**
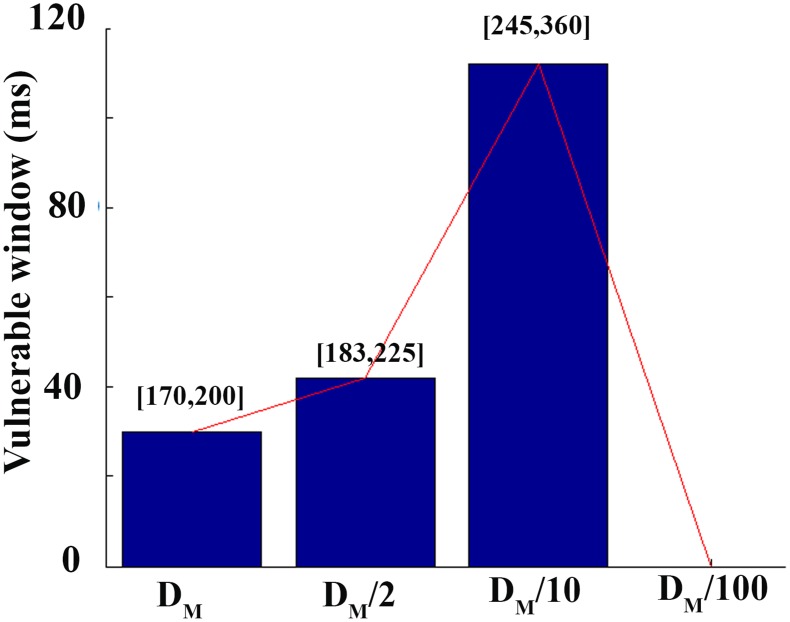
Summary of the computed vulnerable window corresponding to selected reductions inintercellular coupling. Vulnerable window for reentry in ms versus diffusion coefficient in ‘mild fibrosis 2’ configuration (HF ionic remodeling and 28% of fibrosis).

Although intercellular uncoupling led to reentry in normal conditions, we also investigated this effect in failing tissues with defined levels of fibrosis. To determine how the VW was modified by intercellular uncoupling, ‘mild-fibrosis 2’ configuration was adopted and different levels of uncoupling were employed in the simulations. Under such conditions with normal cellular coupling, the VW was 30 ms (see Table 1). When intercellular uncoupling was increased (two-fold decrease of the diffusion coefficient), VW was increased to 42 ms. A ten-fold reduction of conductivity yielded a VW of 112 ms. For very low diffusion coefficients (one hundred-fold reduction), cellular uncoupling was high enough to block the electrical propagation. Thus, as shown in Figure 7, an intermediate level of cellular uncoupling favors reentry generation.

### Analysis of reentry generation in HF with fibrosis using phase maps

**Figure 8:**
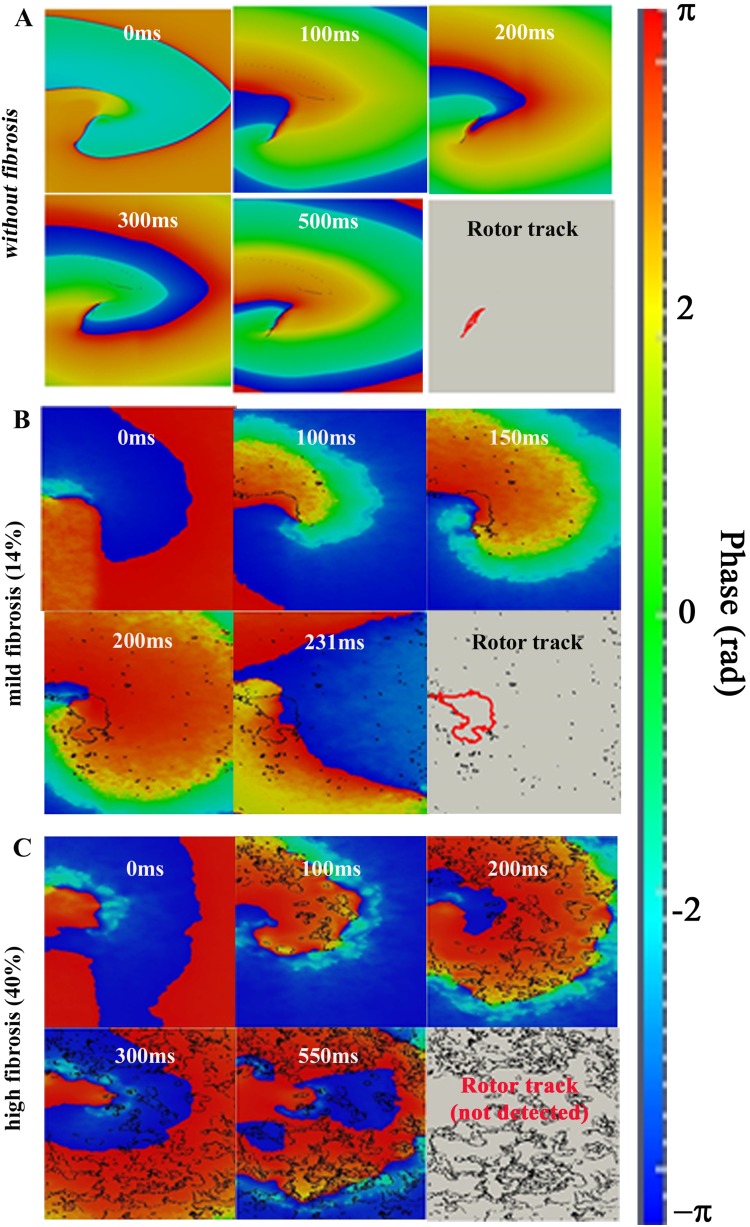
Phase map analysis of evoked rhythm disturbances on the failing human ventricular tissue. Phase maps in electrically remodeled failing tissues assuming selected levels of fibrotic content. In the first case (panel A), ‘without fibrosis’, the intercellular coupling is increased ten-fold. The ‘mild fibrosis’ (panel B) and ‘high fibrosis’ (panel C) cases assume normal intercellular coupling conditions. All transient phase singularities are indicated in black, and the tip of the trajectory is indicated in red in the last snapshot of each panel. For full spatiotemporal evolution of panels A, B, and C, see **Videos S5, S6,** and **S7,** respectively.

To more completely understand the dynamics of reentry in the presence of fibrosis, we employed phase maps, to illustrate and analyze the spatiotemporal organization of reentry dynamics. Figure 8 depicts the evolution of the spiral wave for the selected fibrotic conditions with electrical HF-induced ionic remodeling. When no fibroblasts were present (“without fibrosis” configuration), concentrated phase singularities were detected, revealing clearly the trajectory of the rotor tip. This trajectory can be observed in red color in the last snapshot of panel A. In this setting, reentry developed when intercellular uncoupling was increased. The corresponding spatiotemporal evolution of the instantaneous phase is shown in Video S5. As the fibrotic content of the tissue increased (‘mild fibrosis 2’), under HF electrophysiological remodeling conditions and normal intercellular coupling, the phase singularities were transient and sparse throughout the ventricular tissue. The trajectory of the rotor tip could also be identified (red color in the last snapshot of panel B), although it was less concentrated than in the absence of fibrosis, due to the fibroblast –induced electrical heterogeneities. In this last snapshot all transient phase singularities throughout the simulation time are also shown in black. The corresponding spatiotemporal evolution of the instantaneous phase is shown in Video S6. When a high level of fibrosis was introduced (‘high fibrosis’ configuration), with HF electrophysiological remodeling and normal intercellular coupling, multiple phase singularities appeared during the entire simulation. However, PSs did not follow a specific trajectory. In this case, a rotor tip could not be defined. The phase maps show (in red and blue colors) the phases of the small depolarization, showing only electrotonic interactions, as opposed to AP propagation. The corresponding spatiotemporal evolution of the instantaneous phase is shown in Video S7.

## Discussion

This computational study focussed on the vulnerability to reentry and the dynamics of spiral waves in a virtual failing human ventricular tissue, with transmural heterogeneity, electrophysiological remodeling, cellular uncoupling and fibrosis. It was designed to assess the contributions of each component of heart failure, i.e. ionic remodeling, intercellular uncoupling and fibrosis, to arrhythmogenesis. The principal findings and insights from our work are (i) demonstration that HF electrophysiological remodeling delays the limits of the vulnerable window for reentry but does not alter the width of the VW, so that the likelihood of reentrant activity is not enhanced; (ii) confirmation that fibrosis is determinant for reentry generation; intermediate fibrotic levels increase the VW and favor reentry generation, while high levels hamper it; (iii) evidence that intermediate intercellular uncoupling enhances the VW; (iv) understanding how the active interaction between fibroblasts and failing myocytes increases the presence of phase singularities in ventricular tissues and alters the spiral wave dynamics in reentrant circuits.

### Contribution of electrical remodeling on reentry generation

Patients with severe heart failure are at high risk of sudden cardiac death. In the majority of these patients, sudden cardiac death is due to ventricular tachyarrhythmias (VT). Alterations of the electric properties of single myocytes in heart failure may favor the occurrence of VT in these patients by inducing early or delayed afterdepolarizations (EADs or DADs) [5]. In failing hearts with a healed infarct, VT is caused by reentrant excitation within the network of surviving myocardial fibers in the infarct. In non-ischemic cardiomyopathies, both in humans and in animal models, VT is predominantly due to non-reentrant mechanisms, most likely triggered activity caused by DADs [2], [48]. The generation of EADs and DADs in failing human hearts has also been simulated in computational studies [31], [49] using Grandi et al. AP model [30]. Electrophysiological remodeling in failing hearts not only facilitates triggered activity but it also results in an arrhytmogenic substrate, as shown in the accompanying paper [12]. Both effects are necessary to reentry generation. One of the aims of the present study was to evaluate how HF electrophysiological remodeling affects the probability of reentry generation. Our results suggest that HF electrical remodeling (alone) does not alter the width of the VW with respect to normal conditions, although the limits of the VW are delayed. Indeed, repolarization is delayed in HF and premature stimulation can lead to reentry if applied at later instants of time Moreno et al. [16] evaluated the effects of drugs on reentry generation in a virtual human ventricular tissue with HF-induced electrophysiological remodeling and observed also that conduction block occurred at slower frequencies with HF remodeling, thus delaying the limits of the vulnerable window for reentry generation. This was due to the alteration of cell excitability caused by prolongation of APD in HF. Other computational studies introducing HF electrophysiological remodeling in 2D tissues [17], [18] have been performed but none of these studies focused on the effect of electrical remodeling on vulnerability to reentry, as illustrated in the present study.

### Contribution of structural remodeling on reentry generation

Structural remodeling in the failing heart, i.e. fibrosis and intercellular uncoupling, is an important factor favoring the formation of reentrant waves. Fibrosis is thought to contribute to the deterioration of LV function (impairing the mechanical properties of the left ventricle during diastole) and electrical activity (facilitating arrhythmogenesis) [50], [51]. As shown in the accompanying paper [12], fibrosis decreased conduction velocity in accordance with other studies [4], [11]. Videos S1, S2 and S3 and Figures 3 and 4 show a discontinuous wavefront in the presence of fibrosis as in [20]. Experimentally, Glukhov et al. [11] optically mapped the coronary perfused left ventricular wedge preparations from human hearts with end-stage nonischemic cardiomyopathy and reported nonuniform propagation discontinuities and wave breaks conditioned by strands of increased interstitial fibrosis. It is also known that the excitability of the tissue is altered in the presence of fibroblasts and spontaneous depolarizations were observed in our simulations (see Figure 4). As shown experimentally by Miragoli et al. [52] in tissue cultures, myocytes coupled to fibroblasts might exhibit spontaneous depolarizations, depending on the density of fibrosis and heterocellular coupling conductance (see [53] for review). Similarly, in their simulation study, Greisas and Zlochiver [42] obtained spontaneous excitations. These spontaneous excitations are the result of the myocyte membrane depolarization up to the threshold imposed by the coupled fibroblasts, which exhibit a less negative resting potential. Dispersion of repolarization is also enhanced by structural remodeling [12], setting the substrate for reentry. The alteration of the electrical properties of the tissue in the presence of fibrosis might lead to a higher probability of reentry generation. Indeed, our results suggest that intermediate fibrosis increases the VW and that very high fibrosis hampers reentry generation. A similar biphasic behavior was observed in the computational work of McDowell et al. [21] who simulated reentry in a virtual infarcted rabbit heart for different degrees of fibrosis. Xie et al. [4] also demonstrated that functional fibroblast-myocyte coupling facilitates induction of reentry in a virtual heterogeneous cardiac tissue. Majumder et al. [20] added several levels of diffuse fibrosis with passive interaction with myocytes, in a two dimensional transmural tissue. They reported a variety of non-equilibrium states which led to conduction block and spiral waves; they did not consider the possibility of active interaction between both kind of cells as we did in the present study.

Our results also highlighted the fact that an intermediate level of cellular uncoupling favors reentry generation. This was also reported by Ramirez et al. in the setting of myocardial ischemia (phase 1b) with different intercellular uncouling degrees. Wiegerink et al. [14] also related the increase of intercellular coupling to arrhtyhmia inducibility in a rabbit experimental model of HF.

Thus, structural remodeling strongly modulates the substrate for arrhythmogenesis, as was demonstrated in the accompanying paper [12], and seems to be a determinant factor in reentry generation according to our present results. Although the relationship between the type of reentry and tissue architecture has not been identified, it is likely that in hearts with diffuse fibrosis one or more rotors may maintain the arrhythmia [54] whereby the tissue discontinuities may function as (temporary) anchoring points for the mother rotor [55]. The analysis of the reentrant waves dynamics with structural remodeling deserves in fact special attention.

### Reentrant wave dynamics in the failing heart

During reentry, electrical activity propagates within a defined closed path, thus forming a spiral wave of activation in two dimensions and a scroll wave in three dimensions (3D) [47]. Spiral and scroll waves are active sources of excitation, which organize the spatiotemporal pattern of excitation of the myocardium during arrhythmia. 2D spiral waves are characterized by a wave break at the core of the spiral (spiral wave tip). Gray et al. [56] developed the technique we used in the present study based on phase plane analysis to investigate the dynamics of spiral rotors. They identified phase singularities, and their characterization allowed the tracking of the spiral and its tip dynamics in space, over time [57] and the systematic study of initiation, maintenance and termination of rotors. Wave break and formation of a PS is an essential condition for a rotor to exist. The formation of PS is necessary, although not sufficient, for sustained rotation. In addition, a new excitation wave must be generated. Furthermore, a PS must last over time (at least one rotation period) to form a rotor.

In the present work phase maps analysis was used to assess for the first time the influence of fibrotic content under failing conditions on reentry generation. Our simulation results showed that in the absence of fibrosis the induced reentrant waves presented concentrated and regular rotors; when intermediate levels of fibrosis were considered, the tip of the reentry meandered within a larger area and the vulnerable window was wider. The compactness of the tip trajectory prevents a single spiral from splitting into multiple spirals [19]. Thus, the extent of the fibrosis is a key factor in determining the dynamics of reentrant waves under failing conditions. In the case of a high level of fibrosis only electrotonic cell-cell communication was found, the density of PSs was very high but no tip trajectory could be identified. The amount of PSs in this case gave evidence of the heterogeneous nature of the tissue. The ability to correlate PSs with anatomy provides the potential to investigate the relationship between PSs and structural heterogeneities.

Others have employed phase map analysis to follow the trajectory of the spiral tip and the rotor dynamics during tachycardia or fibrillation [26], [27]. However, little research has been done on the relationship between fibrosis, phase singularities and spiral wave dynamics. Recently Majumder et al. [20] and Nayak et al. [19] performed a dynamical analysis of simulated spiral waves in the presence of fibrosis. However, their study was focussed on the tip trajectory and the non-equilibrium states. A phase analysis was not performed. To our knowledge, the only study analyzing the relationship between simulated fibrosis and phase maps was done by Zlochiver et al. [58]. They demonstrated also that the amount of PSs increased with fibrotic content. However, in their work fibroblasts were considered as passive elements and no heart failure ionic remodeling was considered. In this way, our simulation work complements the above mentioned studies and brings new insights into the effects of heart failure induced electrophysiological and structural remodeling on spiral wave dynamics.

To further characterize the dynamics of spiral waves the spiral wave rotation period was calculated under different conditions of fibrosis (see Figure 5 and table 1) and the rotation frequency slightly decreased with the increase of fibrosis. Zlochiver et al. [58] also observed a decrease in the rotor frequency when the amount of fibrosis was increased. Tanaka et al. [25] reported as well a decrease in the dominant frequency in atrial fibrillation in failing sheep atria and related it to the presence of fibrosis. In a recent study, Greisas et al. [42] calculated the rotation period of the spiral wave, however they used the information of the AP in only one site of the tissue to calculate the period of the rotation wave and found a biphasic behavior: the rotation frequency increased and then decreased when fibrosis was increased.

### Limitations of the study

This study provides novel mechanistic insights into reentry generation in the human failing human heart. We acknowledge, however, that our work has some significant limitations. Firstly, our anatomical description of the cardiac tissue does not include accurate fiber orientation or electrophysiological heterogeneities, as more detailed 3D ventricular models can provide. However, anisotropic conduction was considered in our 2D tissue simulations. Secondly, only the active model of fibroblasts (including ionic currents) was considered as in [21], [32], [39], [59], while others have considered a passive interaction between myocytes and fibroblasts [4], [19], [20], [58]. Although functional coupling between myocytes and fibroblasts has been identified in cultured preparations [60] and ionic currents have been identified in fibroblasts [38], no experiments have reported the presence of active fibroblasts in human. Furthermore, due to the irregular shape of coupled fibroblasts, these are difficult to identify [61]. Thus, modeling and simulation work provides a helpful tool to reproduce this hypothetical scenario. It is also to be noted that a smaller spatial discretization as in [4], would allow a more realistic definition of the size of fibroblasts, taking into account that a fibroblast is more than four times smaller than a myocyte. However, this would increase significantly the computational cost. In any case, in our simulations the ionic models consider the difference of capacity between a fibroblast and a myocyte, scaling accordingly the ionic currents. Finally, with regard to the random introduction of fibroblasts in the tissue, an increase in the number of random configurations for each level of fibrosis would allow to perform a statistical analysis of the effects of fibrosis. Because of the high computational cost this would suppose, we only simulated four different configurations for each case, and it can be stated that the results were very similar.

### Conclusions and clinical implications

In summary, this study shows that electrophysiological and structural remodeling in failing hearts exert an essential influence on reentry generation and dynamics. Electrical coupling of cardiomyocytes with fibroblasts cells alters the anisotropic action potential propagation in the human failing heart in a fashion that significantly depends on the density of fibrotic content and on the degree of intercellular coupling. This raises the possibility that pharmaceutical treatment with eg. rotigaptide, (which increases intercellular coupling), might affect reentrant activity. Phase maps analysis provides a powerful tool to explore abnormal conduction patterns in the setting of ventricular fibrosis. Identifying and studying the different organizational manifestations of phase distribution during arrhythmias in the presence of fibrosis provides one means for objective characterization of arrhythmias. Our findings also may be relevant to ablative procedures, which might have improved success when the targets for ablation enclose the boundaries of tip meandering, as revealed by the phase analysis. Indeed, understanding the relationship between VT organizing centers and fibrosis has important implications for VT ablation since it could provide guidance in determining the optimal targets of VT ablation, as demonstrated by Ashikaga et al. 2013 [62] in their simulation study. They accurately identified optimal targets of ablation in patient-specific heart models and carried out effectively non-invasive ablation. This computational method prior to the clinical procedure supposes a potential translational effort in developing simulation predictions for the optimal targets of VT ablation. Similarly, ablation in zones of complex fractionated electrograms, which are related to the presence of fibrosis [63], and surrounded by phase singularities, has been shown to be effective in simulation studies [64]. Thus, phase maps analysis related to fibrosis seems to be a potent tool prior to the clinical ablation procedures.

## Supporting Information

File S1 (DOCX)

Video S1 **Spiral-wave dynamics in our 2D model of heart failure (HF) in human ventricular myocyte-fibroblast tissue.** This movie shows the evolution of membrane potential (V_m_) in our 2D failing tissue with a fibrotic content of 14.5% (mild fibrosis 1 configuration). The S1–S2 cross-field stimulation protocol was applied (see Methods section for details). Evolution of a spiral wave is shown. 15 frames per second (fps) are used and the movie shows 900 ms of simulation. These results are also shown in Figure 3 panel A.
(RAR)

Video S2 **Ectopic activity in our 2D failing myocyte-fibroblast tissue.** This movie shows the evolution of membrane potential (V_m_) in our 2D failing tissue with a fibrotic content of 28% (mild fibrosis 2 configuration). The differences in resting potential between fibroblasts and myocytes are responsible for the electrotonic interactions between these groups of cells. When the density of fibroblasts is quite high, V_m_ of the adjacent myocytes is depolarized to the action potential (AP) threshold. 15 frames per second (fps) are used and the movie shows 300 ms of simulation. These results are also shown in Figure 4 panel B.
(RAR)

Video S3 **Non reentrant activity our 2D failing myocyte-fibroblast tissue.** This movie shows the evolution of membrane potential (V_m_) in our 2D heart failure (HF) tissue with a fibrotic content of 40% (high fibrosis configuration). The S1–S2 cross-field stimulation protocol was applied (see Methods section for details) and the evolution of electrotonic change in the membrane potential profile is shown. 15 frames per second (fps) are used and the movie shows 800 ms of simulation. These results are also shown in Figure 4 panel D.
(RAR)

Video S4 **Spiral-wave dynamics under conditions of selected levels of intercellular uncoupling.** This movie shows the evolution of membrane potential (V_m_) in our 2D tissue under non electrical remodeling conditions (GPB model) but with structural remodeling included (intercellular resistance ten-fold reduced). S1–S2 cross-field stimulation protocol was applied (see Methods section for details) and the evolution of a spiral wave is shown. 15 frames per second (fps) are used and the movie shows 650 ms of simulation. These results are also shown in Figure 5 panel B.
(RAR)

Video S5 **Phase spatiotemporal evolution without fibrosis.** This movie shows the evolution of the membrane potential (V_m_) (left movie) and the instantaneous phase (right movie) in our 2D tissue with heart failure induced electrical remodeling and intercellular uncoupling (diffusion coefficient was reduced 10 fold). The S1–S2 cross-field stimulation protocol was applied (see Methods section for details). All transient phase singularities are indicated in black; these are maintained in the video to observe their trajectory. 15 frames per second (fps) are used and the movie shows 260 ms of simulation. These results are also shown in Figure 7 panel A.
(RAR)

Video S6 **Phase spatiotemporal evolution with mild fibrosis.** This movie shows the evolution of the membrane potential (V_m_) (left movie) and the instantaneous phase (right movie) in our 2D tissue with heart failure induced electrical remodeling and mild fibrosis. The S1–S2 cross-field stimulation protocol was applied (see Methods section for details). All transient phase singularities are indicated in black; these are maintained in the video to observe their trajectory. 15 frames per second (fps) are used and the movie shows 140 ms of simulation. These results are also shown in Figure 7 panel B.
(RAR)

Video S7 **Phase spatiotemporal evolution with high fibrosis.** This movie shows the evolution of the membrane potential (V_m_) (left movie) and the instantaneous phase (right movie) in our 2D tissue with heart failure induced electrical remodeling and high fibrosis. The S1–S2 cross-field stimulation protocol was applied (see Methods section for details). All transient phase singularities are indicated in black; these are maintained in the video to observe their trajectory. 15 frames per second (fps) are used and the movie shows 425 ms of simulation. These results are also shown in Figure 7 panel C.
(RAR)
