# Correction: Electrophysiological and Structural Remodeling in Heart Failure Modulate Arrhythmogenesis. 2D Simulation Study

**DOI:** 10.1371/journal.pone.0117883

**Published:** 2015-02-06

**Authors:** The PLOS ONE Staff

There are a number of errors in Fig. 4, “Illustration of the effect of fibrosis in human ventricular tissue.” Please see the corrected Fig. 4 here.

**Fig 4:**
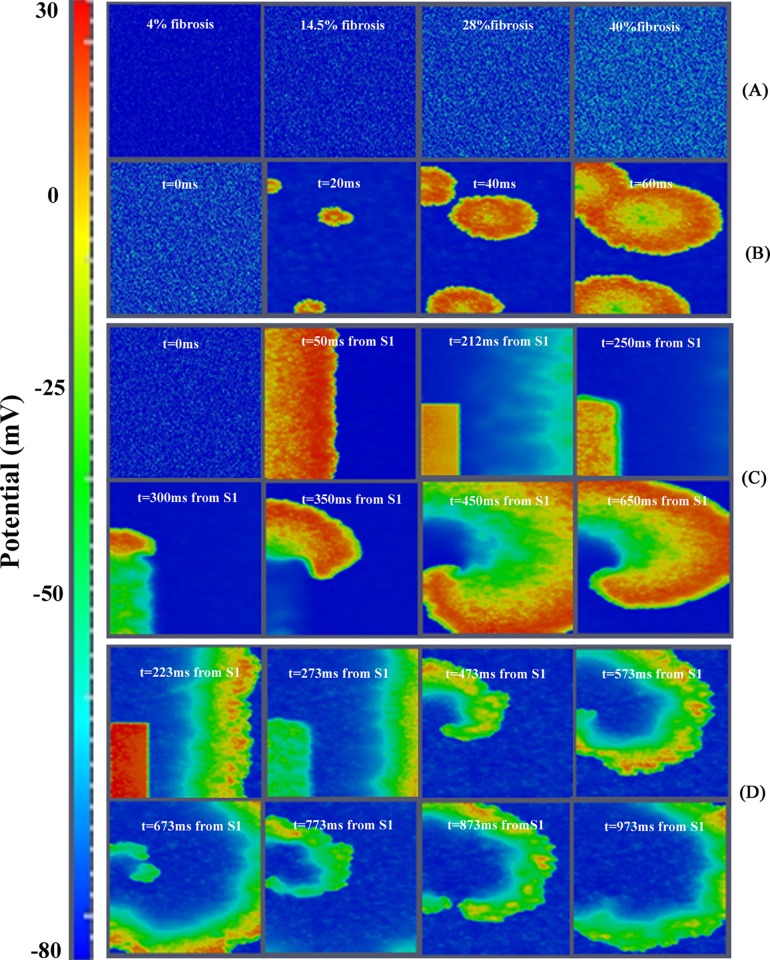
Illustration of the effect of fibrosis in human ventricular tissue. A. Voltage snapshots corresponding to initial conditions, different fibrotic tissue configurations. B. Spontaneous electrical activity in ‘mildfibrosis 2’. C. Voltage snapshots of transmural tissue assuming heart failure conditions together with 28% of random fibrosis (‘mildfibrosis 1’). In the first two frames, propagation of the action potential due to the S1 stimulus is observed and note also that propagation of the action potential elucidated by the S2 stimulus is observed after the third frame. D. Voltage snapshots of transmural tissue assuming heart failure conditions together with 40% of random fibrosis (‘high fibrosis’). S1-S2 cross-field protocol was also used to stimulate the tissue, but in this case only the propagation of stimulus S2 is shown. For full spatiotemporal evolution of panel B see Video S2. For full spatiotemporal evolution of panel D see Video S3.
