# Supplementary material for: Electrophysiological and Structural Remodeling in Heart Failure Modulate Arrhythmogenesis. 2D Simulation Study
Source: PLoS One. 2014 Jul 23;9(7):e103273. doi: 10.1371/journal.pone.0103273 (PMC4108391; doi:10.1371/journal.pone.0103273)
Supplement: File S1 — (DOCX) [file pone.0103273.s001.docx]

**SUPPORTING INFORMATION (S1)**

*RANDOM FIBROSIS CONFIGURATIONS*

The electrical activity of a two dimensional (2D) human cardiac tissue of 5cm x 5cm was simulated containing coupled myocytes and fibroblasts. Heart failure ionic remodeling was applied to myocytes. Fibroblasts distribution was organized randomly, by assigning a probabilistic function. Fibrotic contents of 4%, 14.5%, 28% and 40% were chosen corresponding to the percentage of nodes assigned to the fibroblast ionic model. For each fibrotic content 4 random configurations were simulated and are shown in Figure S1 (fibrotic content of 4%), Figure S2 (fibrotic content of 14.5%), Figure S3 (fibrotic content of 28%) and in Figure S4 (fibrotic content of 4%). Myocytes are represented in red and fibroblasts are represented in blue.

**
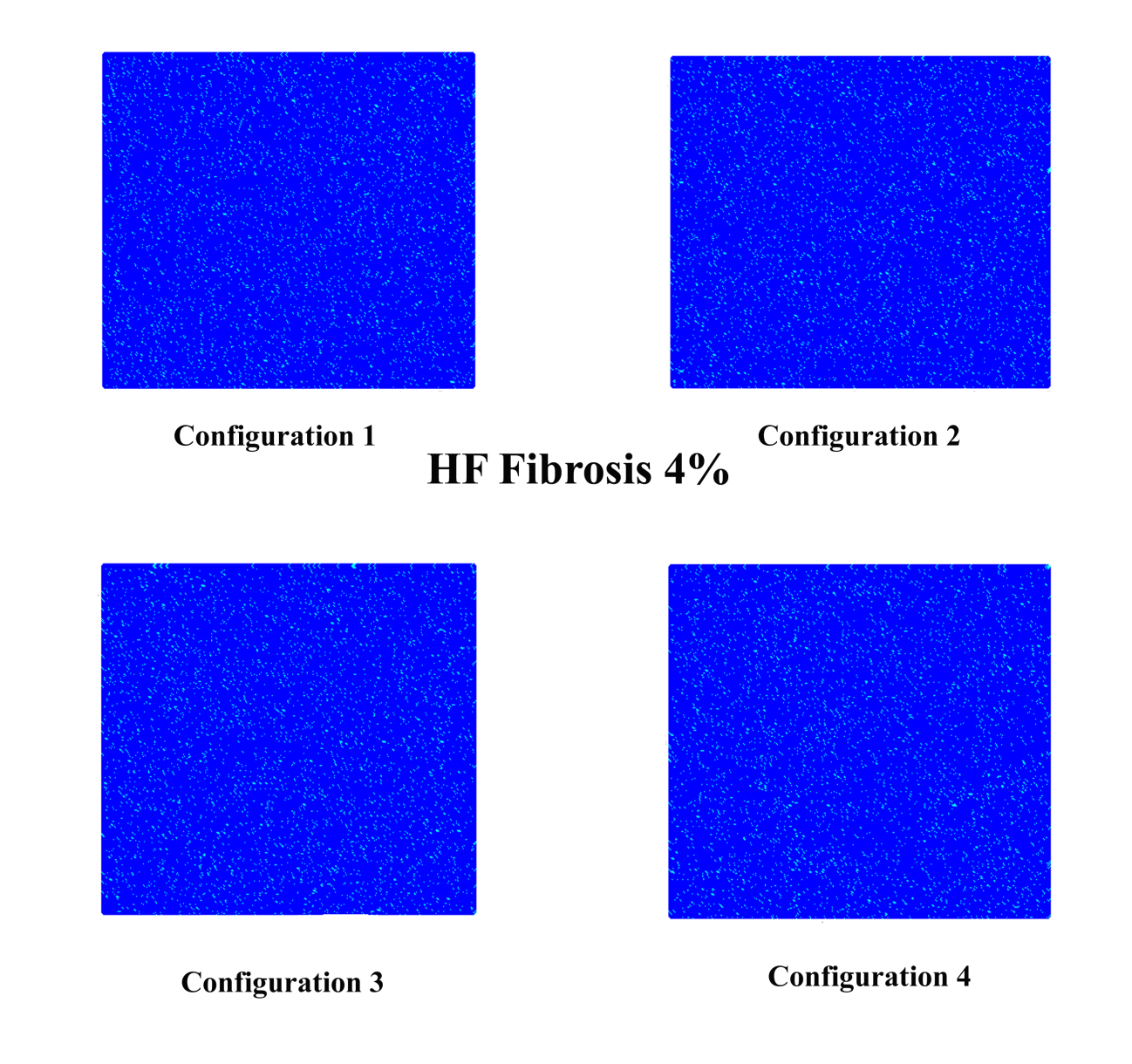
**

**Figure S1.** Random configurations for 4 % fibrosis in the human failing ventricular tissue.

**
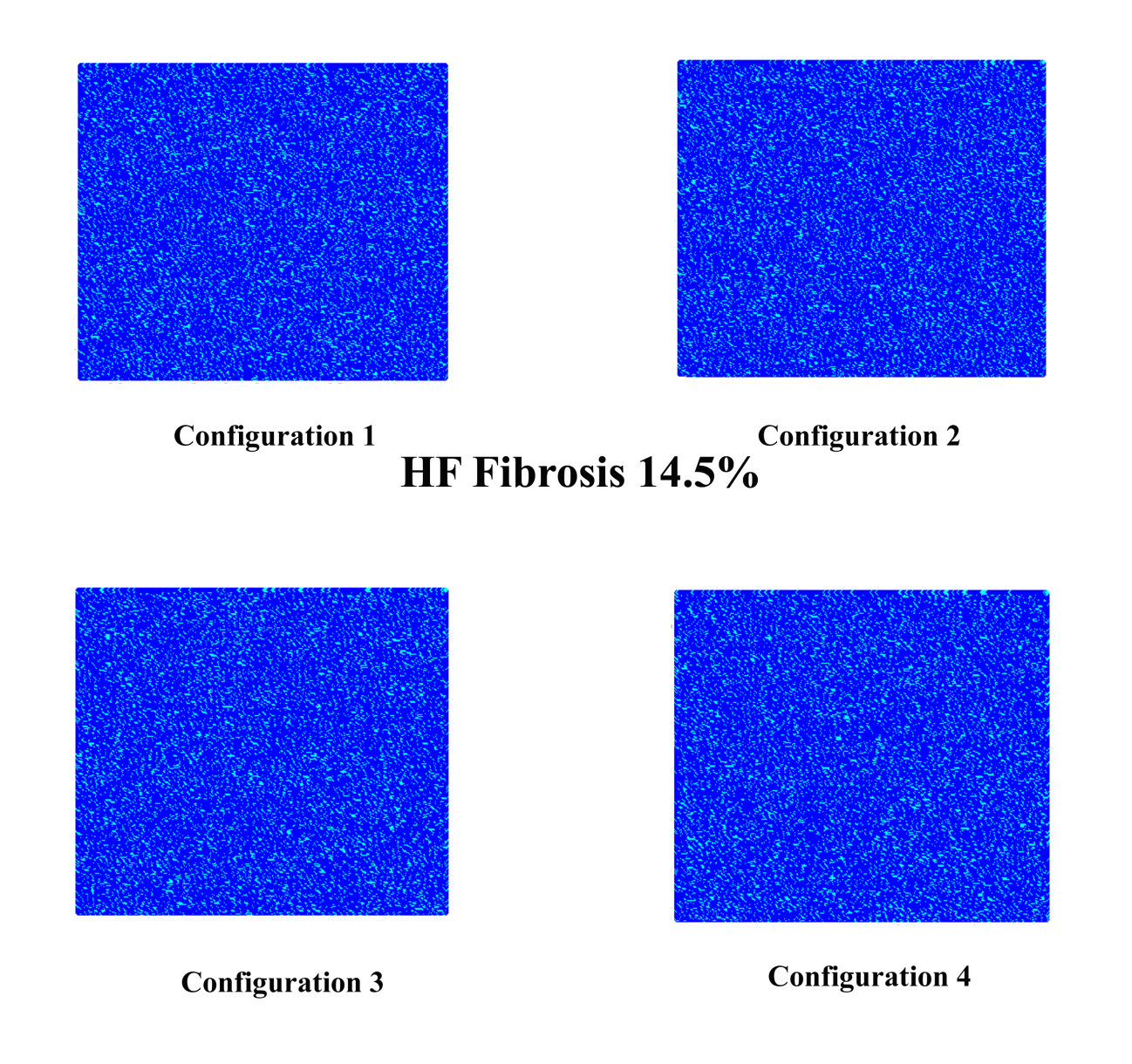
**

**Figure S2.** Random configurations for 14.5 % fibrosis in the human failing ventricular tissue.

**
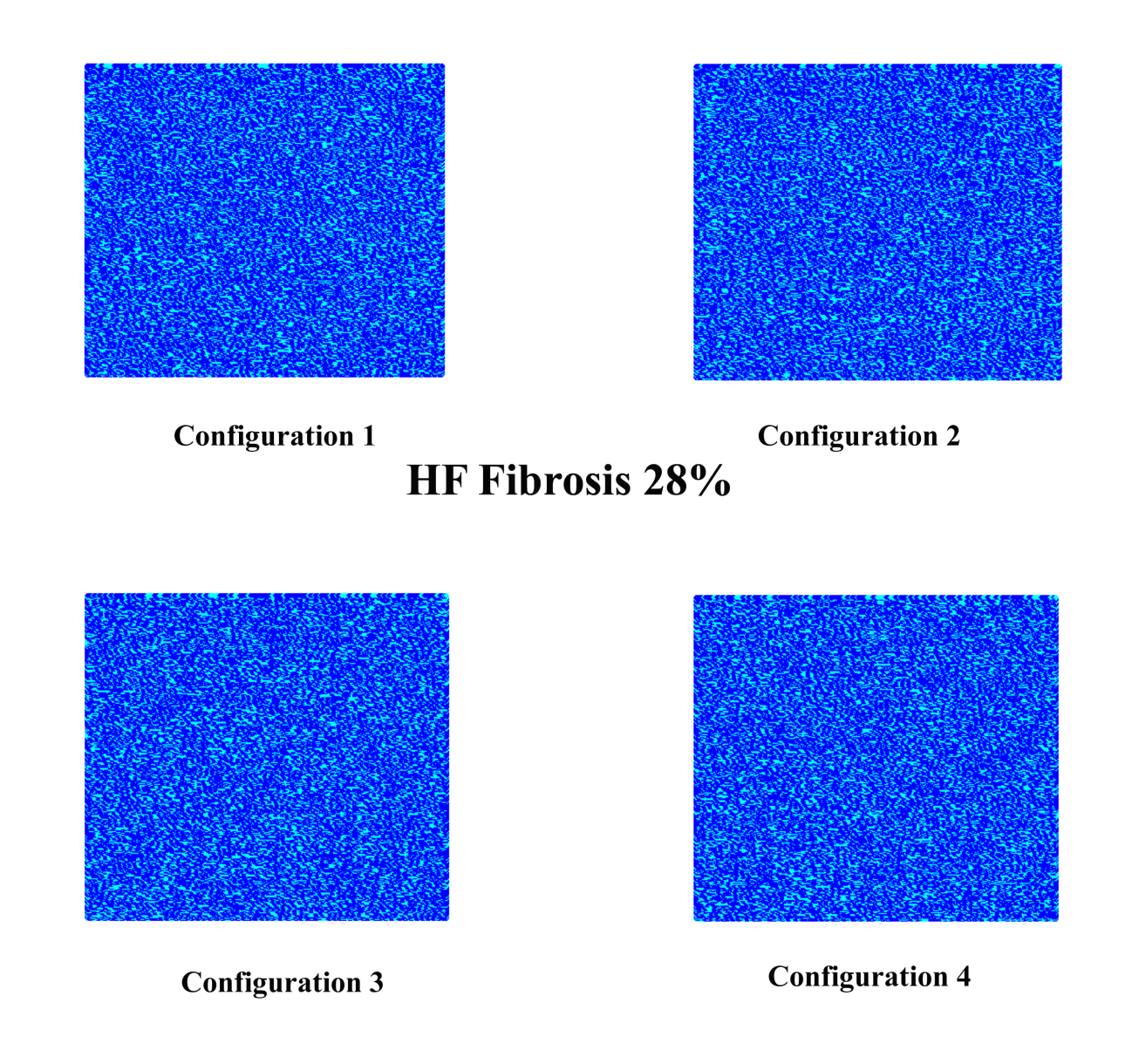
Figure S3.** Random configurations for 28 % fibrosis in the human failing ventricular tissue.

**
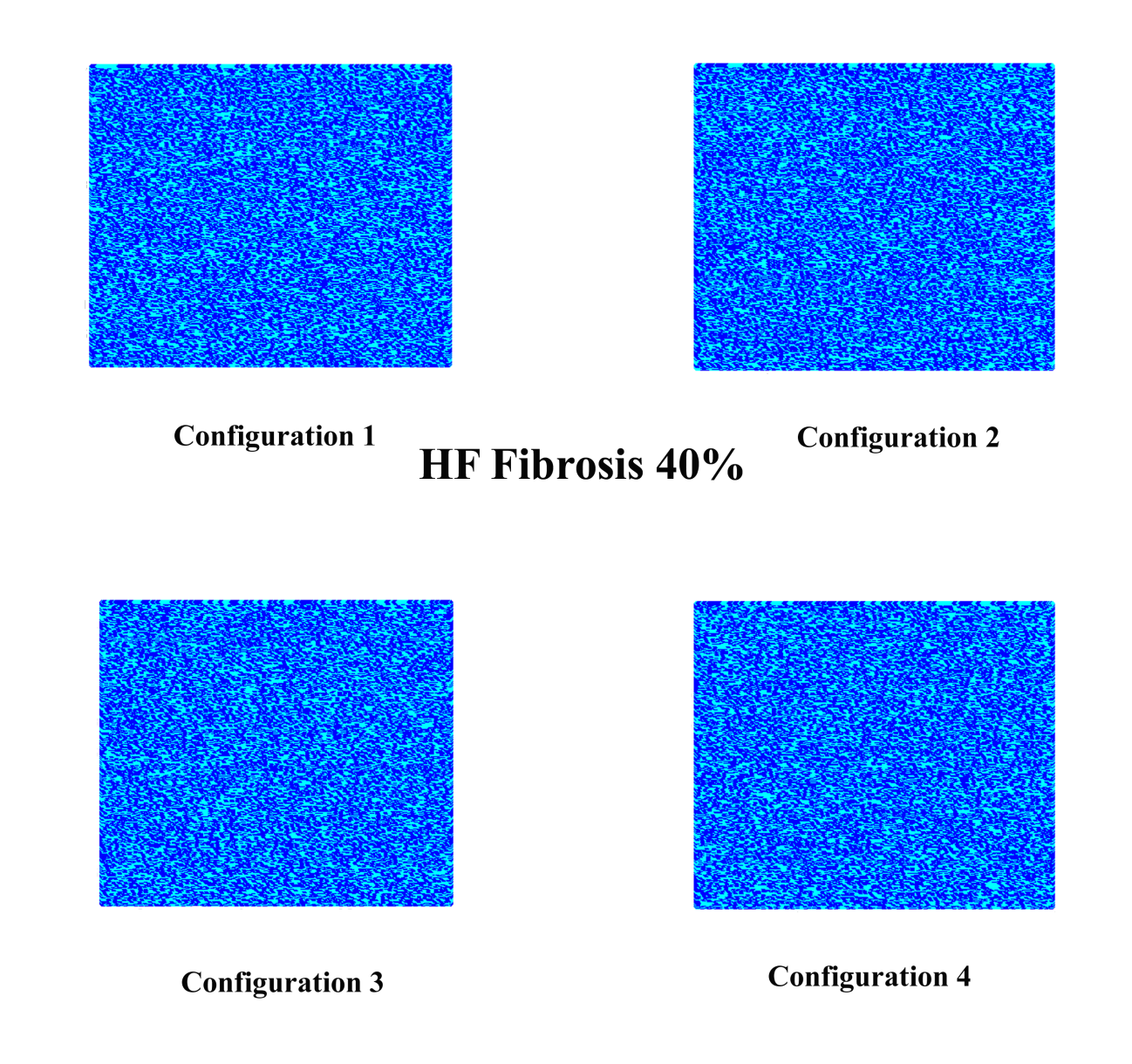
**

**Figure S4.** Random configurations for 40 % fibrosis in the human failing ventricular tissue.

The vulnerable window for reentry was evaluated (as described in the Methods section of the main text) in all the configurations. Table S1 shows the limits and the width of the vulnerable window (VW) in each case. The results of configurations 1 are shown in the main text and Figures of the article. For all the configurations of low fibrosis and high fibrosis no reentry was observed. In the case of mild fibrosis 1, the width of the VW ranged from 20 to 24 ms, and the limits were very similar. When fibrosis increased to mild fibrosis 2, the width of the VW was larger, ranging from 33 to 39 ms.

**Table S1.** Vulnerable window (VW) analysis for different random configurations of fibrosis.

| **Degree of Fibrosis** | **Random Configuration** | **Limits of the VW (ms)** | **Width of the VW (ms)** |
| --- | --- | --- | --- |
| **Low fibrosis (4%)** | 1 | - | 0 |
|  | 2 | - | 0 |
|  | 3 | - | 0 |
|  | 4 | - | 0 |
| **Mild fibrosis 1 (14.5%)** | 1 | [204;224] | 20 |
|  | 2 | [203;223] | 20 |
|  | 3 | [203;225] | 22 |
|  | 4 | [203;227] | 24 |
| **Mild fibrosis 2 (28%)** | 1 | [170;203] | 33 |
|  | 2 | [178;212] | 34 |
|  | 3 | [178;212] | 34 |
|  | 4 | [175;214] | 39 |
| **High fibrosis (40%)** | 1 | - | 0 |
|  | 2 | - | 0 |
|  | 3 | - | 0 |
|  | 4 | - | 0 |
